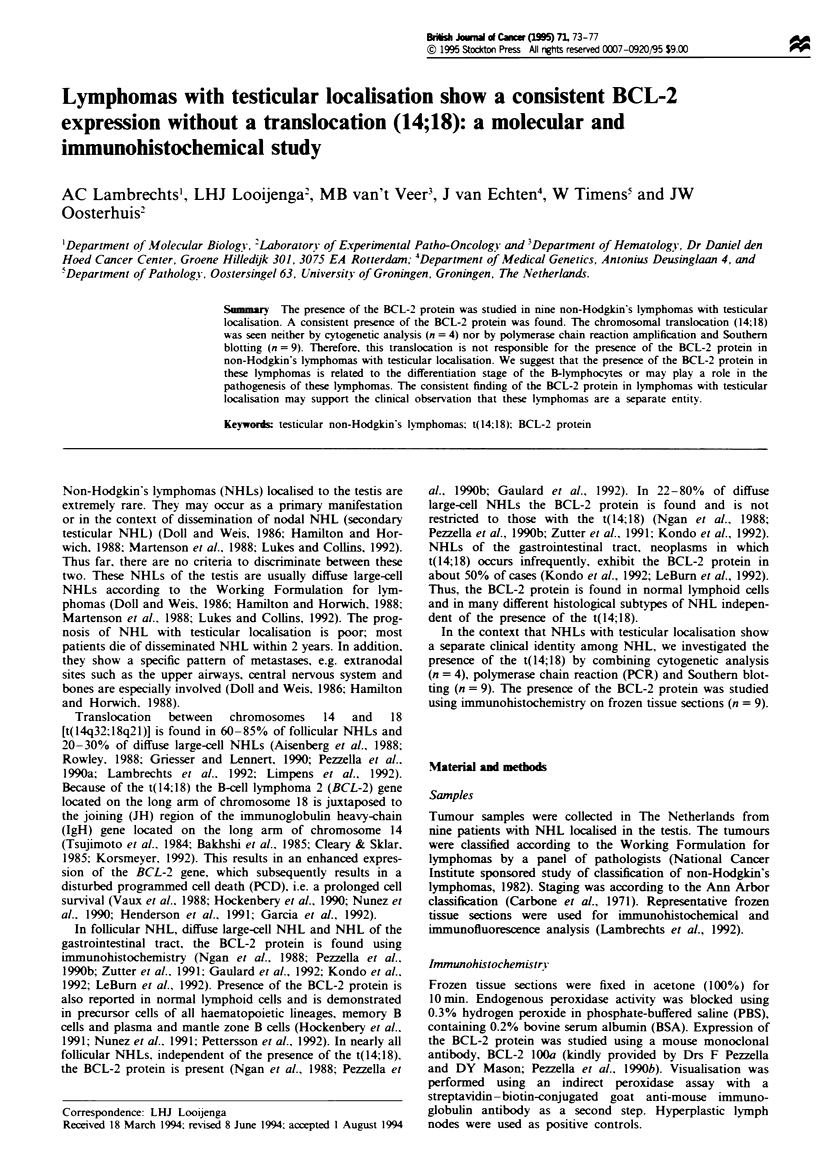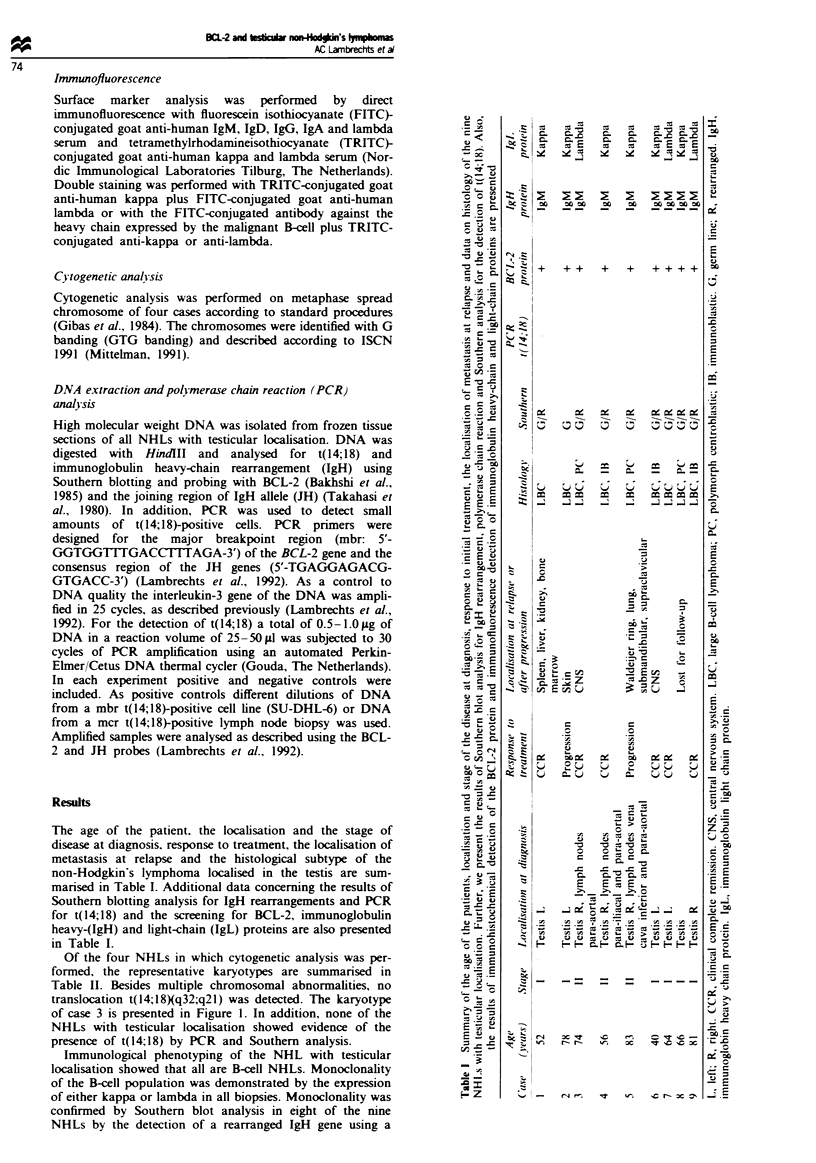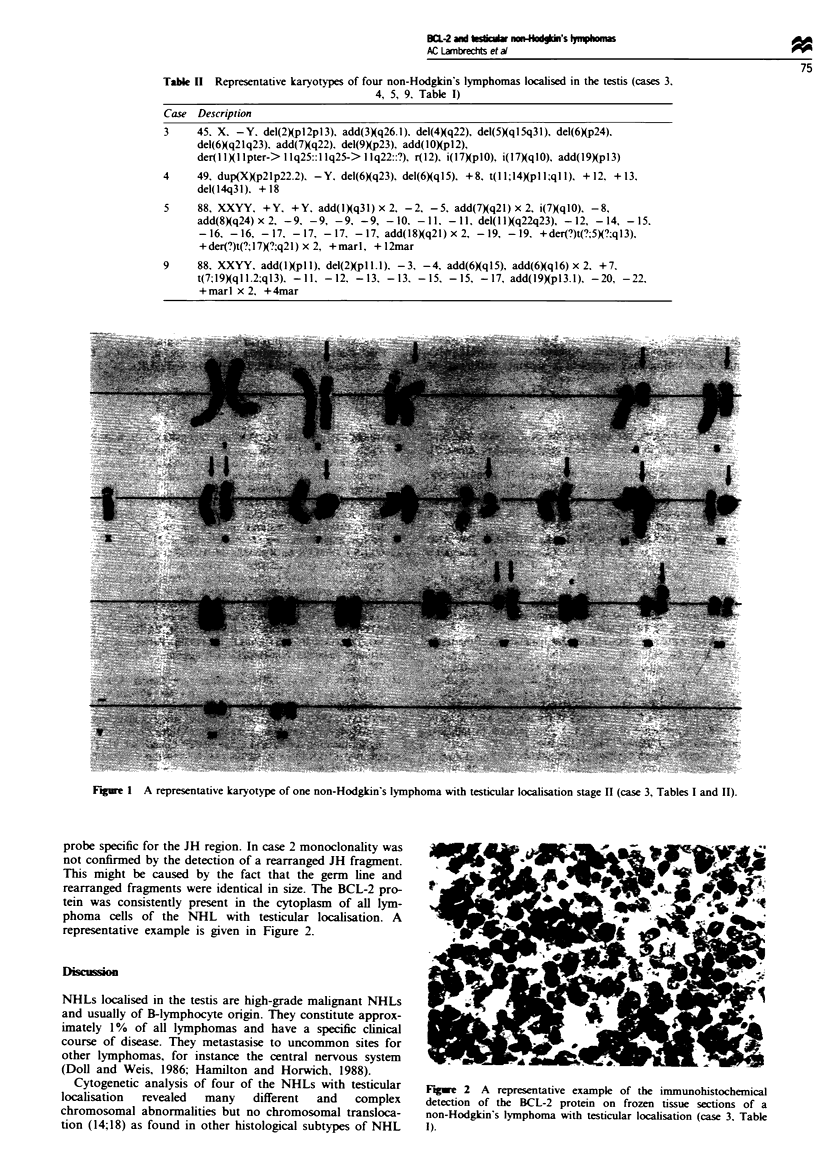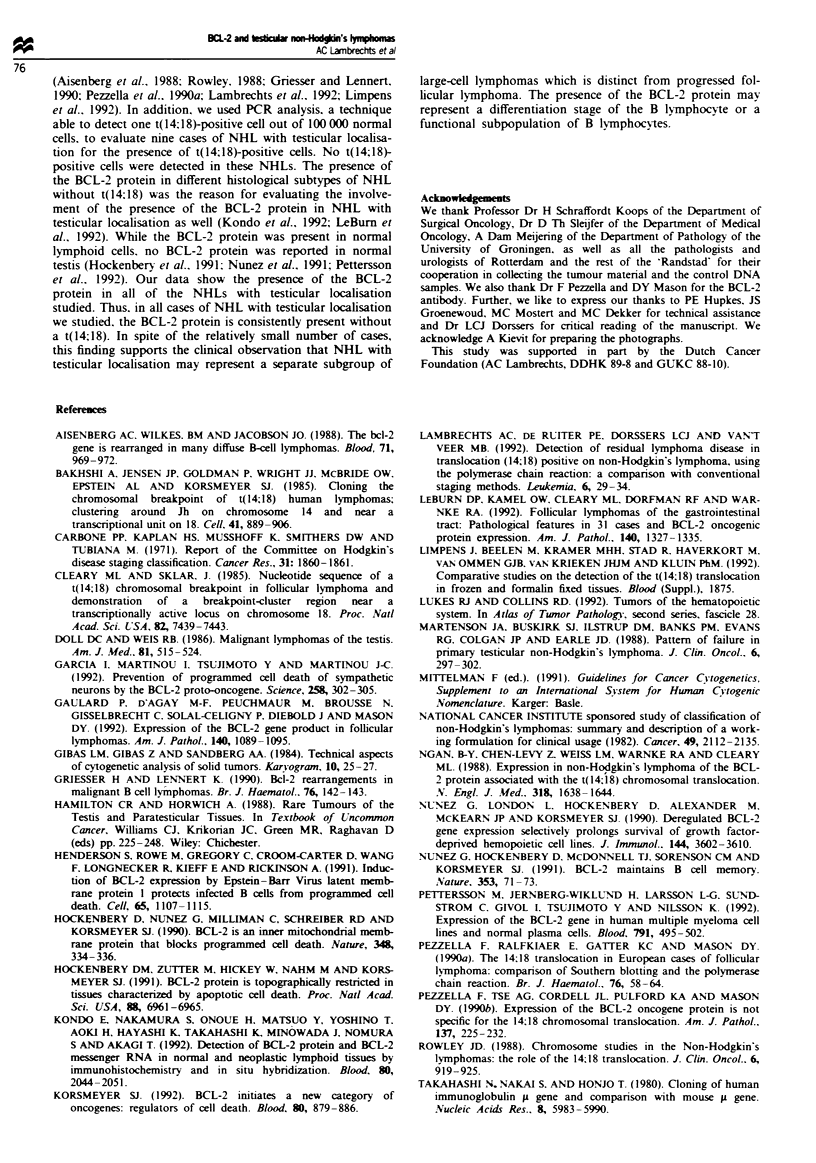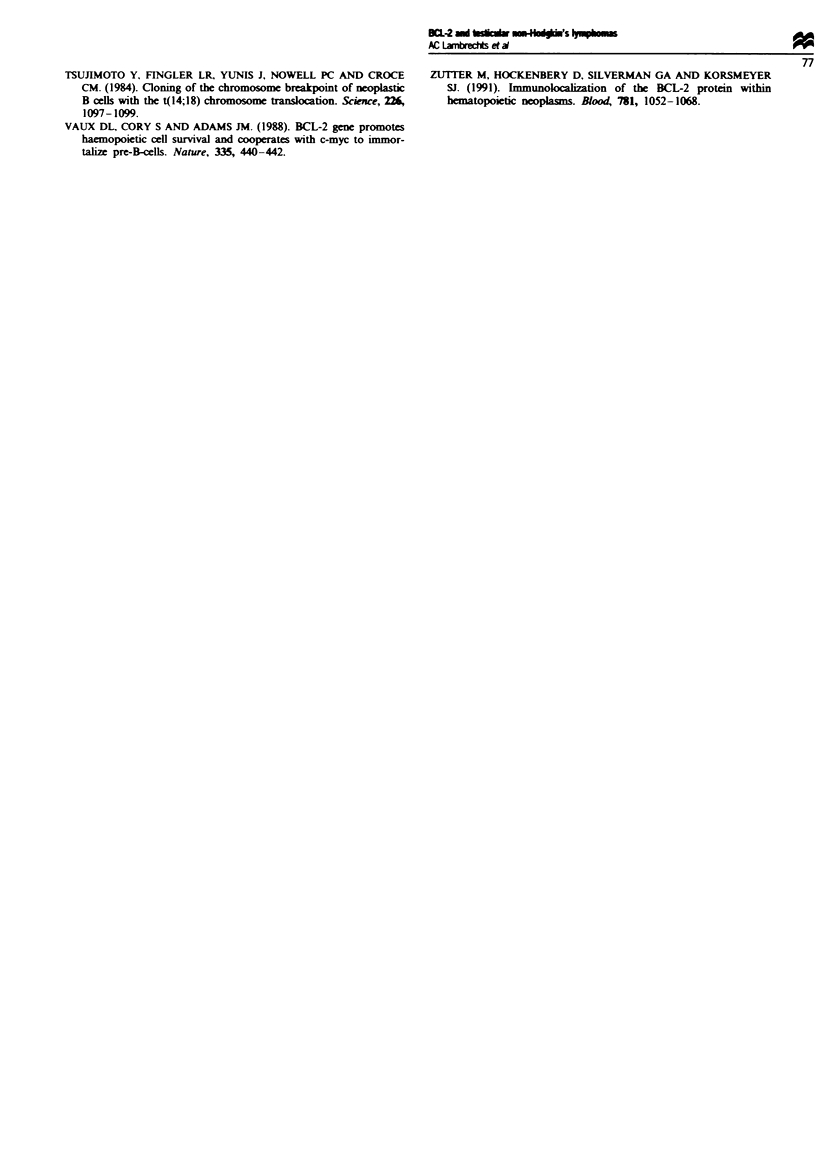# Lymphomas with testicular localisation show a consistent BCL-2 expression without a translocation (14;18): a molecular and immunohistochemical study.

**DOI:** 10.1038/bjc.1995.15

**Published:** 1995-01

**Authors:** A. C. Lambrechts, L. H. Looijenga, M. B. van't Veer, J. van Echten, W. Timens, J. W. Oosterhuis

**Affiliations:** Department of Molecular Biology, Dr Daniel den Hoed Cancer Center, Rotterdam, The Netherlands.

## Abstract

**Images:**


© 1995 Stockton Press All rights reserved 0007-0920/95 $9.00

### Lymphomas with testicular localisation show a consistent BCL-2 expression without a translocation (14;18): a molecular and immunohistochemical study

AC Lambrechts[1], LHJ Looijenga[2], MB van't Veer[3], J van Echten[4], W Timens[5] and JW Oosterhuis[2]

[1]*Department of Molecular Biology,* [2]*Laboratory of Experimental Patho-Oncology and* [3]*Department of Hematology, Dr Daniel den Hoed Cancer Center, Groene Hilledijk 301, 3075 EA Rotterdam;* [4]*Department of Medical Genetics, Antonius Deusinglaan 4, and* [5]*Department of Pathology, Oostersingel 63, University of Groningen, Groningen, The Netherlands.*

**Summary** The presence of the BCL-2 protein was studied in nine non-Hodgkin's lymphomas with testicular localisation. A consistent presence of the BCL-2 protein was found. The chromosomal translocation (14;18) was seen neither by cytogenetic analysis ($n = 4$) nor by polymerase chain reaction amplification and Southern blotting ($n = 9$). Therefore, this translocation is not responsible for the presence of the BCL-2 protein in non-Hodgkin's lymphomas with testicular localisation. We suggest that the presence of the BCL-2 protein in these lymphomas is related to the differentiation stage of the B-lymphocytes or may play a role in the pathogenesis of these lymphomas. The consistent finding of the BCL-2 protein in lymphomas with testicular localisation may support the clinical observation that these lymphomas are a separate entity.

**Keywords:** testicular non-Hodgkin's lymphomas; t(14;18); BCL-2 protein

Non-Hodgkin's lymphomas (NHLs) localised to the testis are extremely rare. They may occur as a primary manifestation or in the context of dissemination of nodal NHL (secondary testicular NHL) (Doll and Weis, 1986; Hamilton and Horwich, 1988; Martenson *et al.*, 1988; Lukes and Collins, 1992). Thus far, there are no criteria to discriminate between these two. These NHLs of the testis are usually diffuse large-cell NHLs according to the Working Formulation for lymphomas (Doll and Weis, 1986; Hamilton and Horwich, 1988; Martenson *et al.*, 1988; Lukes and Collins, 1992). The prognosis of NHL with testicular localisation is poor; most patients die of disseminated NHL within 2 years. In addition, they show a specific pattern of metastases, e.g. extranodal sites such as the upper airways, central nervous system and bones are especially involved (Doll and Weis, 1986; Hamilton and Horwich, 1988).

Translocation between chromosomes 14 and 18 [t(14q32;18q21)] is found in 60-85% of follicular NHLs and 20-30% of diffuse large-cell NHLs (Aisenberg *et al.*, 1988; Rowley, 1988; Griesser and Lennert, 1990; Pezzella *et al.*, 1990a; Lambrechts *et al.*, 1992; Limpens *et al.*, 1992). Because of the t(14;18) the B-cell lymphoma 2 (*BCL*-2) gene located on the long arm of chromosome 18 is juxtaposed to the joining (JH) region of the immunoglobulin heavy-chain (IgH) gene located on the long arm of chromosome 14 (Tsujimoto *et al.*, 1984; Bakhshi *et al.*, 1985; Cleary & Sklar, 1985; Korsmeyer, 1992). This results in an enhanced expression of the *BCL*-2 gene, which subsequently results in a disturbed programmed cell death (PCD), i.e. a prolonged cell survival (Vaux *et al.*, 1988; Hockenbery *et al.*, 1990; Nunez *et al.*, 1990; Henderson *et al.*, 1991; Garcia *et al.*, 1992).

In follicular NHL, diffuse large-cell NHL and NHL of the gastrointestinal tract, the BCL-2 protein is found using immunohistochemistry (Ngan *et al.*, 1988; Pezzella *et al.*, 1990b; Zutter *et al.*, 1991; Gaulard *et al.*, 1992; Kondo *et al.*, 1992; LeBurn *et al.*, 1992). Presence of the BCL-2 protein is also reported in normal lymphoid cells and is demonstrated in precursor cells of all haematopoietic lineages, memory B cells and plasma and mantle zone B cells (Hockenbery *et al.*, 1991; Nunez *et al.*, 1991; Pettersson *et al.*, 1992). In nearly all follicular NHLs, independent of the presence of the t(14;18), the BCL-2 protein is present (Ngan *et al.*, 1988; Pezzella *et al.*, 1990b; Gaulard *et al.*, 1992). In 22-80% of diffuse large-cell NHLs the BCL-2 protein is found and is not restricted to those with the t(14;18) (Ngan *et al.*, 1988; Pezzella *et al.*, 1990b; Zutter *et al.*, 1991; Kondo *et al.*, 1992). NHLs of the gastrointestinal tract, neoplasms in which t(14;18) occurs infrequently, exhibit the BCL-2 protein in about 50% of cases (Kondo *et al.*, 1992; LeBurn *et al.*, 1992). Thus, the BCL-2 protein is found in normal lymphoid cells and in many different histological subtypes of NHL independent of the presence of the t(14;18).

In the context that NHLs with testicular localisation show a separate clinical identity among NHL, we investigated the presence of the t(14;18) by combining cytogenetic analysis ($n = 4$), polymerase chain reaction (PCR) and Southern blotting ($n = 9$). The presence of the BCL-2 protein was studied using immunohistochemistry on frozen tissue sections ($n = 9$).

### Material and methods

### Samples

Tumour samples were collected in The Netherlands from nine patients with NHL localised in the testis. The tumours were classified according to the Working Formulation for lymphomas by a panel of pathologists (National Cancer Institute sponsored study of classification of non-Hodgkin's lymphomas, 1982). Staging was according to the Ann Arbor classification (Carbone *et al.*, 1971). Representative frozen tissue sections were used for immunohistochemical and immunofluorescence analysis (Lambrechts *et al.*, 1992).

### Immunohistochemistry

Frozen tissue sections were fixed in acetone (100%) for 10 min. Endogenous peroxidase activity was blocked using 0.3% hydrogen peroxide in phosphate-buffered saline (PBS), containing 0.2% bovine serum albumin (BSA). Expression of the BCL-2 protein was studied using a mouse monoclonal antibody, BCL-2 100*a* (kindly provided by Drs F Pezzella and DY Mason; Pezzella *et al.*, 1990*b*). Visualisation was performed using an indirect peroxidase assay with a streptavidin-biotin-conjugated goat anti-mouse immunoglobulin antibody as a second step. Hyperplastic lymph nodes were used as positive controls.

Correspondence: LHJ Looijenga
Received 18 March 1994; revised 8 June 1994; accepted 1 August 1994

*Immunofluorescence*

Surface marker analysis was performed by direct immunofluorescence with fluorescein isothiocyanate (FITC)-conjugated goat anti-human IgM, IgD, IgG, IgA and lambda serum and tetramethylrhodamineisothiocyanate (TRITC)-conjugated goat anti-human kappa and lambda serum (Nordic Immunological Laboratories Tilburg, The Netherlands). Double staining was performed with TRITC-conjugated goat anti-human kappa plus FITC-conjugated goat anti-human lambda or with the FITC-conjugated antibody against the heavy chain expressed by the malignant B-cell plus TRITC-conjugated anti-kappa or anti-lambda.

*Cytogenetic analysis*

Cytogenetic analysis was performed on metaphase spread chromosome of four cases according to standard procedures (Gibas *et al.*, 1984). The chromosomes were identified with G banding (GTG banding) and described according to ISCN 1991 (Mittelman, 1991).

*DNA extraction and polymerase chain reaction (PCR) analysis*

High molecular weight DNA was isolated from frozen tissue sections of all NHLs with testicular localisation. DNA was digested with *Hind*III and analysed for t(14;18) and immunoglobulin heavy-chain rearrangement (IgH) using Southern blotting and probing with BCL-2 (Bakhshi *et al.*, 1985) and the joining region of IgH allele (JH) (Takahasi *et al.*, 1980). In addition, PCR was used to detect small amounts of t(14;18)-positive cells. PCR primers were designed for the major breakpoint region (mbr: 5'-GGTGGTTTGACCTTTAGA-3') of the *BCL*-2 gene and the consensus region of the JH genes (5'-TGAGGAGACG-GTGACC-3') (Lambrechts *et al.*, 1992). As a control to DNA quality the interleukin-3 gene of the DNA was amplified in 25 cycles, as described previously (Lambrechts *et al.*, 1992). For the detection of t(14;18) a total of 0.5–1.0 μg of DNA in a reaction volume of 25–50 μl was subjected to 30 cycles of PCR amplification using an automated Perkin-Elmer/Cetus DNA thermal cycler (Gouda, The Netherlands). In each experiment positive and negative controls were included. As positive controls different dilutions of DNA from a mbr t(14;18)-positive cell line (SU-DHL-6) or DNA from a mcr t(14;18)-positive lymph node biopsy was used. Amplified samples were analysed as described using the BCL-2 and JH probes (Lambrechts *et al.*, 1992).

### Results

The age of the patient, the localisation and the stage of disease at diagnosis, response to treatment, the localisation of metastasis at relapse and the histological subtype of the non-Hodgkin's lymphoma localised in the testis are summarised in Table I. Additional data concerning the results of Southern blotting analysis for IgH rearrangements and PCR for t(14;18) and the screening for BCL-2, immunoglobulin heavy-(IgH) and light-chain (IgL) proteins are also presented in Table I.

Of the four NHLs in which cytogenetic analysis was performed, the representative karyotypes are summarised in Table II. Besides multiple chromosomal abnormalities, no translocation t(14;18)(q32;q21) was detected. The karyotype of case 3 is presented in Figure 1. In addition, none of the NHLs with testicular localisation showed evidence of the presence of t(14;18) by PCR and Southern analysis.

Immunological phenotyping of the NHL with testicular localisation showed that all are B-cell NHLs. Monoclonality of the B-cell population was demonstrated by the expression of either kappa or lambda in all biopsies. Monoclonality was confirmed by Southern blot analysis in eight of the nine NHLs by the detection of a rearranged IgH gene using a

**Table II** Representative karyotypes of four non-Hodgkin's lymphomas localised in the testis (cases 3, 4, 5, 9, Table I)

| *Case* | *Description* |
|---|---|
| 3 | 45, X, −Y, del(2)(p12p13), add(3)(q26.1), del(4)(q22), del(5)(q15q31), del(6)(p24), del(6)(q21q23), add(7)(q22), del(9)(p23), add(10)(p12), der(11)(11pter->11q25::11q25->11q22::?), r(12), i(17)(p10), i(17)(q10), add(19)(p13) |
| 4 | 49, dup(X)(p21p22.2), −Y, del(6)(q23), del(6)(q15), +8, t(11;14)(p11;q11), +12, +13, del(14q31), +18 |
| 5 | 88, XXYY, +Y, +Y, add(1)(q31) × 2, −2, −5, add(7)(q21) × 2, i(7)(q10), −8, add(8)(q24) × 2, −9, −9, −9, −9, −10, −11, −11, del(11)(q22q23), −12, −14, −15, −16, −16, −17, −17, −17, −17, add(18)(q21) × 2, −19, −19, +der(?)t(?;5)(?;q13), +der(?)t(?;17)(?;q21) × 2, +mar1, +12mar |
| 9 | 88, XXYY, add(1)(p11), del(2)(p11.1), −3, −4, add(6)(q15), add(6)(q16) × 2, +7, t(7;19)(q11.2;q13), −11, −12, −13, −13, −15, −15, −17, add(19)(p13.1), −20, −22, +mar1 × 2, +4mar |

**Figure 1** A representative karyotype of one non-Hodgkin's lymphoma with testicular localisation stage II (case 3, Tables I and II).

probe specific for the JH region. In case 2 monoclonality was not confirmed by the detection of a rearranged JH fragment. This might be caused by the fact that the germ line and rearranged fragments were identical in size. The BCL-2 protein was consistently present in the cytoplasm of all lymphoma cells of the NHL with testicular localisation. A representative example is given in Figure 2.

### Discussion

NHLs localised in the testis are high-grade malignant NHLs and usually of B-lymphocyte origin. They constitute approximately 1% of all lymphomas and have a specific clinical course of disease. They metastasise to uncommon sites for other lymphomas, for instance the central nervous system (Doll and Weis, 1986; Hamilton and Horwich, 1988).

Cytogenetic analysis of four of the NHLs with testicular localisation revealed many different and complex chromosomal abnormalities but no chromosomal translocation (14;18) as found in other histological subtypes of NHL

**Figure 2** A representative example of the immunohistochemical detection of the BCL-2 protein on frozen tissue sections of a non-Hodgkin's lymphoma with testicular localisation (case 3, Table I).

(Aisenberg et al., 1988; Rowley, 1988; Griesser and Lennert, 1990; Pezzella et al., 1990a; Lambrechts et al., 1992; Limpens et al., 1992). In addition, we used PCR analysis, a technique able to detect one t(14;18)-positive cell out of 100 000 normal cells, to evaluate nine cases of NHL with testicular localisation for the presence of t(14;18)-positive cells. No t(14;18)-positive cells were detected in these NHLs. The presence of the BCL-2 protein in different histological subtypes of NHL without t(14;18) was the reason for evaluating the involvement of the presence of the BCL-2 protein in NHL with testicular localisation as well (Kondo et al., 1992; LeBurn et al., 1992). While the BCL-2 protein was present in normal lymphoid cells, no BCL-2 protein was reported in normal testis (Hockenbery et al., 1991; Nunez et al., 1991; Pettersson et al., 1992). Our data show the presence of the BCL-2 protein in all of the NHLs with testicular localisation studied. Thus, in all cases of NHL with testicular localisation we studied, the BCL-2 protein is consistently present without a t(14;18). In spite of the relatively small number of cases, this finding supports the clinical observation that NHL with testicular localisation may represent a separate subgroup of large-cell lymphomas which is distinct from progressed follicular lymphoma. The presence of the BCL-2 protein may represent a differentiation stage of the B lymphocyte or a functional subpopulation of B lymphocytes.

**Acknowledgements**
We thank Professor Dr H Schraffordt Koops of the Department of Surgical Oncology, Dr D Th Sleijfer of the Department of Medical Oncology, A Dam Meijering of the Department of Pathology of the University of Groningen, as well as all the pathologists and urologists of Rotterdam and the rest of the 'Randstad' for their cooperation in collecting the tumour material and the control DNA samples. We also thank Dr F Pezzella and DY Mason for the BCL-2 antibody. Further, we like to express our thanks to PE Hupkes, JS Groenewoud, MC Mostert and MC Dekker for technical assistance and Dr LCJ Dorssers for critical reading of the manuscript. We acknowledge A Kievit for preparing the photographs.

This study was supported in part by the Dutch Cancer Foundation (AC Lambrechts, DDHK 89-8 and GUKC 88-10).

**References**

AISENBERG AC, WILKES, BM AND JACOBSON JO. (1988). The bcl-2 gene is rearranged in many diffuse B-cell lymphomas. *Blood*, **71**, 969–972.

BAKHSHI A, JENSEN JP, GOLDMAN P, WRIGHT JJ, MCBRIDE OW, EPSTEIN AL AND KORSMEYER SJ. (1985). Cloning the chromosomal breakpoint of t(14;18) human lymphomas; clustering around Jh on chromosome 14 and near a transcriptional unit on 18. *Cell*, **41**, 889–906.

CARBONE PP, KAPLAN HS, MUSSHOFF K, SMITHERS DW AND TUBIANA M. (1971). Report of the Committee on Hodgkin's disease staging classification. *Cancer Res.*, **31**: 1860–1861.

CLEARY ML AND SKLAR, J. (1985). Nucleotide sequence of a t(14;18) chromosomal breakpoint in follicular lymphoma and demonstration of a breakpoint-cluster region near a transcriptionally active locus on chromosome 18. *Proc. Natl Acad. Sci. USA*, **82**, 7439–7443.

DOLL DC AND WEIS RB. (1986). Malignant lymphomas of the testis. *Am. J. Med.*, **81**, 515–524.

GARCIA I, MARTINOU I, TSUJIMOTO Y AND MARTINOU J-C. (1992). Prevention of programmed cell death of sympathetic neurons by the BCL-2 proto-oncogene. *Science*, **258**, 302–305.

GAULARD P, D'AGAY M-F, PEUCHMAUR M, BROUSSE N, GISSELBRECHT C, SOLAL-CELIGNY P, DIEBOLD J AND MASON DY. (1992). Expression of the BCL-2 gene product in follicular lymphomas. *Am. J. Pathol.*, **140**, 1089–1095.

GIBAS LM, GIBAS Z AND SANDBERG AA. (1984). Technical aspects of cytogenetic analysis of solid tumors. *Karyogram*, **10**, 25–27.

GRIESSER H AND LENNERT K. (1990). Bcl-2 rearrangements in malignant B cell lymphomas. *Br. J. Haematol.*, **76**, 142–143.

HAMILTON CR AND HORWICH A. (1988). Rare Tumours of the Testis and Paratesticular Tissues. In *Textbook of Uncommon Cancer*, Williams CJ, Krikorian JC, Green MR, Raghavan D (eds) pp. 225–248. Wiley: Chichester.

HENDERSON S, ROWE M, GREGORY C, CROOM-CARTER D, WANG F, LONGNECKER R, KIEFF E AND RICKINSON A. (1991). Induction of BCL-2 expression by Epstein–Barr Virus latent membrane protein 1 protects infected B cells from programmed cell death. *Cell*, **65**, 1107–1115.

HOCKENBERY D, NUNEZ G, MILLIMAN C, SCHREIBER RD AND KORSMEYER SJ. (1990). BCL-2 is an inner mitochondrial membrane protein that blocks programmed cell death. *Nature*, **348**, 334–336.

HOCKENBERY DM, ZUTTER M, HICKEY W, NAHM M AND KORSMEYER SJ. (1991). BCL-2 protein is topographically restricted in tissues characterized by apoptotic cell death. *Proc. Natl Acad. Sci. USA*, **88**, 6961–6965.

KONDO E, NAKAMURA S, ONOUE H, MATSUO Y, YOSHINO T, AOKI H, HAYASHI K, TAKAHASHI K, MINOWADA J, NOMURA S AND AKAGI T. (1992). Detection of BCL-2 protein and BCL-2 messenger RNA in normal and neoplastic lymphoid tissues by immunohistochemistry and in situ hybridization. *Blood*, **80**, 2044–2051.

KORSMEYER SJ. (1992). BCL-2 initiates a new category of oncogenes: regulators of cell death. *Blood*, **80**, 879–886.

LAMBRECHTS AC, DE RUITER PE, DORSSERS LCJ AND VAN'T VEER MB. (1992). Detection of residual lymphoma disease in translocation (14;18) positive on non-Hodgkin's lymphoma, using the polymerase chain reaction: a comparison with conventional staging methods. *Leukemia*, **6**, 29–34.

LEBURN DP, KAMEL OW, CLEARY ML, DORFMAN RF AND WARNKE RA. (1992). Follicular lymphomas of the gastrointestinal tract: Pathological features in 31 cases and BCL-2 oncogenic protein expression. *Am. J. Pathol.*, **140**, 1327–1335.

LIMPENS J, BEELEN M, KRAMER MHH, STAD R, HAVERKORT M, VAN OMMEN GJB, VAN KRIEKEN JHJM AND KLUIN PhM. (1992). Comparative studies on the detection of the t(14;18) translocation in frozen and formalin fixed tissues. *Blood* (Suppl.), 1875.

LUKES RJ AND COLLINS RD. (1992). Tumors of the hematopoietic system. In *Atlas of Tumor Pathology*, second series, fascicle 28.

MARTENSON JA, BUSKIRK SJ, ILSTRUP DM, BANKS PM, EVANS RG, COLGAN JP AND EARLE JD. (1988). Pattern of failure in primary testicular non-Hodgkin's lymphoma. *J. Clin. Oncol.*, **6**, 297–302.

MITTELMAN F (ed.). (1991). *Guidelines for Cancer Cytogenetics, Supplement to an International System for Human Cytogenic Nomenclature*. Karger: Basle.

NATIONAL CANCER INSTITUTE sponsored study of classification of non-Hodgkin's lymphomas: summary and description of a working formulation for clinical usage (1982). *Cancer*, **49**, 2112–2135.

NGAN, B-Y, CHEN-LEVY Z, WEISS LM, WARNKE RA AND CLEARY ML. (1988). Expression in non-Hodgkin's lymphoma of the BCL-2 protein associated with the t(14;18) chromosomal translocation. *N. Engl. J. Med.*, **318**, 1638–1644.

NUNEZ G, LONDON L, HOCKENBERY D, ALEXANDER M, MCKEARN JP AND KORSMEYER SJ. (1990). Deregulated BCL-2 gene expression selectively prolongs survival of growth factor-deprived hemopoietic cell lines. *J. Immunol.*, **144**, 3602–3610.

NUNEZ G, HOCKENBERY D, MCDONNELL TJ, SORENSON CM AND KORSMEYER SJ. (1991). BCL-2 maintains B cell memory. *Nature*, **353**, 71–73.

PETTERSSON M, JERNBERG-WIKLUND H, LARSSON L-G, SUNDSTROM C, GIVOL I, TSUJIMOTO Y AND NILSSON K. (1992). Expression of the BCL-2 gene in human multiple myeloma cell lines and normal plasma cells. *Blood*, **791**, 495–502.

PEZZELLA F, RALFKIAER E, GATTER KC AND MASON DY. (1990a). The 14;18 translocation in European cases of follicular lymphoma: comparison of Southern blotting and the polymerase chain reaction. *Br. J. Haematol.*, **76**, 58–64.

PEZZELLA F, TSE AG, CORDELL JL, PULFORD KA AND MASON DY. (1990b). Expression of the BCL-2 oncogene protein is not specific for the 14;18 chromosomal translocation. *Am. J. Pathol.*, **137**, 225–232.

ROWLEY JD. (1988). Chromosome studies in the Non-Hodgkin's lymphomas: the role of the 14;18 translocation. *J. Clin. Oncol.*, **6**, 919–925.

TAKAHASHI N, NAKAI S, AND HONJO T. (1980). Cloning of human immunoglobulin $\mu$ gene and comparison with mouse $\mu$ gene. *Nucleic Acids Res.*, **8**, 5983–5990.

TSUJIMOTO Y, FINGLER LR, YUNIS J, NOWELL PC AND CROCE CM. (1984). Cloning of the chromosome breakpoint of neoplastic B cells with the t(14;18) chromosome translocation. *Science*, **226**, 1097–1099.

VAUX DL, CORY S AND ADAMS JM. (1988). BCL-2 gene promotes haemopoietic cell survival and cooperates with c-myc to immortalize pre-B-cells. *Nature*, **335**, 440–442.

ZUTTER M, HOCKENBERY D, SILVERMAN GA AND KORSMEYER SJ. (1991). Immunolocalization of the BCL-2 protein within hematopoietic neoplasms. *Blood*, **781**, 1052–1068.